# Graph Multihead Attention Pooling with Self-Supervised Learning

**DOI:** 10.3390/e24121745

**Published:** 2022-11-29

**Authors:** Yu Wang, Liang Hu, Yang Wu, Wanfu Gao

**Affiliations:** College of Computer Science and Technology, Jilin University, Changchun 130012, China

**Keywords:** network analysis, graph neural networks, graph multihead attention, self-supervised learning

## Abstract

Graph neural networks (GNNs), which work with graph-structured data, have attracted considerable attention and achieved promising performance on graph-related tasks. While the majority of existing GNN methods focus on the convolutional operation for encoding the node representations, the graph pooling operation, which maps the set of nodes into a coarsened graph, is crucial for graph-level tasks. We argue that a well-defined graph pooling operation should avoid the information loss of the local node features and global graph structure. In this paper, we propose a hierarchical graph pooling method based on the multihead attention mechanism, namely GMAPS, which compresses both node features and graph structure into the coarsened graph. Specifically, a multihead attention mechanism is adopted to arrange nodes into a coarsened graph based on their features and structural dependencies between nodes. In addition, to enhance the expressiveness of the cluster representations, a self-supervised mechanism is introduced to maximize the mutual information between the cluster representations and the global representation of the hierarchical graph. Our experimental results show that the proposed GMAPS obtains significant and consistent performance improvements compared with state-of-the-art baselines on six benchmarks from the biological and social domains of graph classification and reconstruction tasks.

## 1. Introduction

Over the past years, deep neural networks have achieved great success in various tasks such as computer vision [1], natural language processing [2], and video processing [3]. However, with the development of data science, the form of data is no longer limited to Euclidean-based data. Ubiquitous non-Euclidean graph data have entered the focus of researchers, such as social networks [4,5,6], biological networks [7,8], knowledge graphs [9], etc. Therefore, GNNs, which extend deep neural network techniques to graph data, have become a research hotspot of great interest [10,11,12]. In recent years, a myriad of GNN methods have been proposed by researchers, which can be categorized into two groups: spectral methods [13,14,15] and spatial methods [5,16]. For spectral methods, they have their theoretical basis in the field of graph signal processing and introduce filters as graph convolution for the purpose of noise reduction of graph signals. For spatial methods, graph convolution is defined by the connection of nodes in topological space, where feature information is directly transferred from one node to its neighbors. Due to their convincing performance and interpretability, GNNs have been widely applied to graph-related tasks, such as node classification [5,17,18], link prediction [19,20], and graph classification [13,21].

Although the majority of the existing work focuses on the design of convolutional operations, graph pooling, which maps nodes into a coarsened graph, is critical for capturing hierarchical structural information in graph classification tasks. However, unlike the pooling operations in traditional CNNs, graph pooling operations are challenging due to the fact that graphs usually contain diverse irregular topologies and no explicit spatial sequence of nodes [21]. A simple graph pooling operation is globally summing or averaging the representations of the nodes in the entire graph to generate a graph-level representation [16]. However, such a pooling operation simply treats all elements as equal, which ignores the differences in the relative importance of various node features. Furthermore, the inherently flat structure of the pooling operation restricts the ability to hierarchically represent the whole graph. To address these limitations, several differentiable hierarchical pooling methods [21,22,23,24] have been proposed to cluster or sample nodes in the graph layer by layer with a neural network architecture in an end-to-end scheme. However, all those methods have obvious drawbacks. In the node sampling approaches, each pooling layer unnecessarily discards a certain percentage of nodes, which causes unnecessary loss of node features and destroys the inherent substructure in the graph. In the node clustering approaches, the two main parts are clustering assignment and constructing a coarsened graph structure. However, these current heuristic-based linear clustering assignment approaches [21,25] are not adaptive to accurately learning the graph representation in a specific downstream task, so have room for improvement. We contend that unnecessary node discarding and ineffective coarsened graph creation are barely adequate because a well-defined graph pooling operation should prevent the loss of node properties and graph topology information.

In this work, to obtain an accurate representation of the graph, we needed design a graph pooling method that can compress and encode the node set into a coarsened graph, and construct the coarsened graph structure. To this end, following DiffPool [21], the graph pooling problem was considered as a node clustering assignment problem, where each cluster represents a node in the next-layer coarsened graph. Specifically, we divided the graph pooling problem into three main units: node clustering assignment, coarsened graph construction, and self-supervised mutual information module. First, to overcome the inability of simple neural network models to discriminate important nodes, a multihead attention mechanism [26] was adopted as the main component in the pooling operation. Second, the clustering assignment matrix could be approximated by the product of *Q* and *K* regarding the attention mechanism, which constructs the structure of the coarsened graph. In addition, to ensure the expressive ability of cluster representations, we introduced a self-supervised mechanism [27,28] to maximize the mutual information between cluster representations and the global representation of the hierarchical graph. By using these three units as basic modules, graph multihead attention pooling with self-supervised learning (GMAPS) was designed, which compresses the given node features and graph structure into a coarsened graph, encodes the clusters (nodes) in the coarsened graph, and constructs the coarsened graph structure. Finally, we experimentally validated the performance of GMAPS in a graph classification task on six datasets from the biological and social domains, where the proposed method outperformed seven state-of-the-art baselines. Our main contributions are summarized as follows:

- We considered the graph pooling as a node clustering assignment problem. For accurate clustering assignment, a multihead attention mechanism based on GNN was introduced to sufficiently consider the connections between nodes in terms of features and the structure. Then, we derived *Q* and *K* in the attention model to generate the topology of the coarsened graph.
- Self-supervised learning was adopted to maximize the mutual information between the cluster representations and hierarchically global representations, which further optimized the node representation in the coarsened graph.
- Finally, the experimental results showed that the proposed GMAPS significantly outperformed baseline methods in the graph classification task on six publicly available datasets from the biological and social domains.

The rest of this paper is organized as follows: In Section 2, we briefly review the related studies about GNNs and graph pooling. Section 3 introduces some preliminaries and presents the details of our proposed model. In Section 4, experimental results on six benchmark datasets are shown and analyzed to highlight the benefits. Finally, we conclude the paper in Section 5.

## 2. Related Work

### 2.1. Graph Neural Networks

Existing GNNs models generally utilize the message-passing strategy [29] to encode node representations and have achieved promising performance on node classification [4], link prediction [30], and graph classification [31] tasks. Advances are often categorized into two branches: spectral approaches [13,14,15] and spatial approaches [5,16]. For spectral approaches, the graph convolutional operation is typically defined according to graph spectral theory. Ref. [32] suggested that a spectral filter can be approximated by a truncated expansion in terms of Chebyshev *K*-order polynomials of graph Laplacian. Later, ChebNet [15] used this *K*-localized convolution to define a convolutional neural network on graphs. The graph convolutional network (GCN) [5] further limits the *K*-localized convolution to K=1 as the layer-wise convolutional operation and implements rich convolutional filter functions by stacking multiples of such layers [33]. To jointly consider the local and global consistency on graphs, deep graph CNN (DGCNN) [34] extended GCN by adding a convolutional operation with positive point-wise mutual information (PPMI) matrix. For spatial approaches, the convolutional operation typically directly aggregates the neighborhood information to the central node. Specifically, [4] proposed GraphSAGE, a general inductive framework that encodes representations by sampling a fixed-size set of local neighborhoods and aggregating their features by mean, LSTM, or pooling. GAT [17] incorporates the attention mechanism into the aggregation step and utilizes the self-attention strategy to assign different weights to aggregated neighborhoods. Inspired by GAT, many researchers have incorporated structured information to the transformer by developing the structure-aware self-attention mechanism [35,36,37]. However, to the bet of our knowledge, there is no previous study applying a transformer to graph pooling operations. More details about graph neural networks can be found in several comprehensive reviews [38,39,40].

### 2.2. Graph Pooling

Graph pooling is an essential unit in the hierarchical graph representation learning task, which captures node features and the hierarchical graph structure. Directly averaging or summing the node representations of the entire graph is the simplest pooling operation; however, it ignores the diverse weight of nodes and the hierarchical graph structure [16]. Existing graph pooling approaches can be broadly categorized into node sampling and node clustering approaches. For the node sampling approaches, they score the nodes by various mechanisms and then proportionally select the nodes with high scores as the nodes of the coarsened graph, while the nodes with low scores are discarded. gPool [22] employs a trainable projection vector to adaptively downsample a subset of nodes, yet ignores the graph structure information. To integrate node features and graph topology in the graph pooling layer, SAGPool [23] utilizes graph convolution to compute self-attention scores. Furthermore, ASAP [41] defines the local neighbors within a fixed receptive field as clusters, and then exploits an attention mechanism to compute the fitness scores based on local extremum information. However, this branching approach inevitably loses some important node feature information and graph topology information while discarding nodes. In contrast, node clustering approaches replace the node sampling as a fixed number of clusters with aggregated nodes. DiffPool [21] proposes a differentiable hierarchical clustering module by training an assignment matrix and a topology matrix of coarsened graphs. In order to achieve satisfactory performance, it appends an auxiliary link prediction objective and entropy regularization. Yet, this heuristic model cannot be adapted to match specific downstream tasks. MinCutPool [25] formulates a continuous relaxation of the normalized minCUT problem [42] based on the theory of spectral clustering and trains an adaptive clustering assignment by optimizing this objective function. However, it involves high computational complexity and many iterations.

## 3. Proposed Model

The key idea of the proposed GMAPS is that it enables the construction of graph pooling through a differentiable node assignment based on a multihead attention mechanism and a hierarchical objective based on maximizing mutual information. In this section, we present the overall GNN architecture and show the details of each module.

### 3.1. Preliminaries

A graph *G* is represented as (V,E), where $V=\{v_{0},v_{1}\ldots ,v_{n}\}$ and $E=\{e_{0},e_{1},\ldots ,e_{l}\}$ are the setd of nodes and edges, respectively; *n* and *l* denote the number of nodes and edges, respectively. Let $A\in {\left\{0,1\right\}}^{n\times n}$ denote the adjacent matrix and $X\in R^{n\times f}$ be the node feature matrix, where *f* is the dimension of node features. Given a set of labeled graphs $G=\{\left(G_{1},y_{1}\right),\left(G_{2},y_{2}\right),\cdots \}$ where $y_{i}\in Y$ indicates the label corresponding to graph $G_{i}\in G$, the goal of the graph classification task is to learn a mapping F:G→Y that maps the set of graphs to the set of labels. In addition, the nodes (clusters) and the graph structure are changed after each pooling layer, so we further denote the adjacent matrix and hidden representation matrix of graph $G_{i}$ fed into the *k*th layer as $A_{i}^{k}\in R^{n_{i}^{k}\times n_{i}^{k}}$ and $H_{i}^{k}\in R^{n_{i}^{k}\times d}$, respectively, where *d* is the dimension of any hidden representations in neural networks, and $n_{i}^{k}$ means the number of nodes in $G_{i}$ at layer *k*.

#### 3.1.1. Graph Neural Networks

GNNs learn node representations through various aggregation schemes, which are generally described as the following message-passing architecture [29]:

(1) $$H^{k+1}=\gamma^{k}\;\left(H^{k},\;\varphi^{k}\;\left(H^{k},\;A^{k}\right)\right),$$

where γ(·) denotes a differentiable update function, and φ(·) denotes a differentiable, permutation-invariant aggregation function, e.g., sum, mean, or max. $H^{k}$ is the node representation fed into the *k*th layer. The aggregation function φ(·) aggregates the representations of neighboring nodes into an aggregated representation. Then, the update function γ(·) concatenates (or sums) the current node representations with the aggregated representations as the updated node representations. Particularly, the input node representation $H^{1}$ is initialized using the node features on the graph, e.g., $H^{1}=X$.

There are many possible implementations for the GNN architecture, such as GCN [5], GraphSAGE [4], and GAT [17]. In this study, we implemented the pooling operations on top of the GCN architecture, due to its classical and efficient nature. For the (k+1)th layer in GCN, the message-passing architecture can be formalized as follows:

(2) $$H^{k+1}=\mathrm{ReLU}\left({\tilde{D}}^{-\frac{1}{2}}\tilde{A}{\tilde{D}}^{-\frac{1}{2}}H^{k}W^{k}\right),$$

where ReLU(·) is the nonlinear activation function, $\tilde{A}=A+I$ is the adjacent matrix with self-loops, ${\tilde{D}}_{ii}=\sum_{j}{\tilde{A}}_{ij}$ is the diagonal degree matrix, and $W^{k}\in R^{d\times d}$ is a trainable weight matrix.

#### 3.1.2. Graph Pooling

GNNs are inherently flat, as they only propagate information across the edge of a graph. The goal of graph pooling is to define a differentiable end-to-end operation to generate the structure and the representation of a coarsened graph for hierarchical GNN models. Following Ying’s strategy [21], the pooling problem can be considered as using the output of the GNN module to learn how to cluster or sample nodes to generate a coarsened graph so that we can use this coarsened graph as the input to the next layer. We denote the learned clustering assignment matrix at *k*th pooling layer as $S^{k}\in R^{n^{k}\times n^{k+1}}$, and the node (cluster) representations $H^{k+1}$ and adjacent matrix $A^{k+1}$ of the coarsened graph of the k+1 layer can be generated as follows:

(3) $$H^{k+1}={S^{k}}^{⊤}H^{k}\in R^{n^{k+1}\times d},$$

(4) $$A^{k+1}={S^{k}}^{⊤}A^{k}S^{k}\in R^{n^{k+1}\times n^{k+1}},$$

where $\cdot^{⊤}$ denotes the matrix transpose. Equation (3) uses the clustering assignment $S^{k}$ to softly cluster the *k*th layer nodes $H^{k}$ to generate the coarsened clusters (nodes) $H^{k+1}$ at k+1 layer. Similarly, Equation (4) generates the adjacent matrix $A^{k+1}$ of the coarsened graph of the k+1 layer to represent the connection strength between clusters (nodes).

### 3.2. Overall Neural Network Architecture

**Figure 1:**
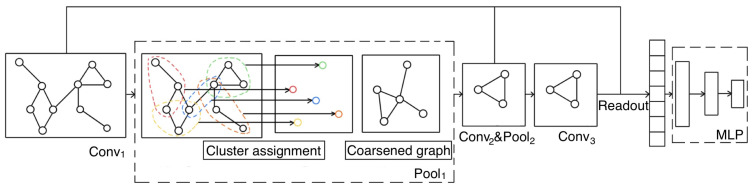
The overall neural network architecture of proposed GMAPS.

Figure 1 illustrates the framework of the proposed graph multihead attention pooling with self-supervised learning (GMAPS), which is implemented interleaved with graph convolutional operations to build a stacking GNNs architecture. GMAPS can be divided into three major components as node clustering assignment, coarsened graph construction, and self-supervised mutual information module. The whole GNNs architecture generally alternately stacks multiple graph convolutional layers and graph pooling layers in a hierarchical fashion. Then, a readout function is used to aggregate and concatenate the representations of each convolutional layer to generate the graph-level representation. Finally, the graph-level representations are fed into a multilayer perceptron (MLP) for the graph classification task. In the following, we detail each of the three major components of the proposed pooling method.

### 3.3. Node Clustering Assignment

Existing methods [21,25] generally utilize GNNs and MLPs to learn node clustering assignment; however, the former ignores the internal connections between nodes, and the latter ignores nonlinear relationships, which limit the performance of the clustering. In this study, to overcome these limitations, the attention mechanism was adopted to learn node clustering assignments.

Let $n_{c}$ denote the number of next-layer clusters (nodes), and we have queries $Q\in R^{n_{c}\times d}$, keys $K\in R^{n\times d}$ and values $V\in R^{n\times d}$ as the input. The scaled dot-product attention [26] function can be formalized as follows:

(5) $$\mathrm{Attention}\left(Q,\;K,\;V\right)=\mathrm{softmax}\left(\frac{QK^{⊤}}{\sqrt{d}}\right)V.$$

In this paper, queries Q and keys K denote clusters and nodes, respectively. The dot-product operation $QK^{⊤}$ calculates the correlation between them, followed by a normalized function softmax(·). Finally, based on the normalized weights, a weighted summation of the values V of the nodes is performed to obtain the representations of clusters.

Furthermore, instead of performing a single attention function with *d*-dimensional queries, keys, and values, we can linearly separate the queries, keys, and values with *h* different learned projections function into d/h dimensions. Multihead attention allows the model to jointly attend to information from different representation subspaces. The output of the multihead attention function is computed as follows:

(6) $$\begin{array}{c} \mathrm{MultiHead}\left(Q,K,V\right)=\mathrm{Concat}\left({\mathrm{head}}_{1},\ldots ,{\mathrm{head}}_{h}\right)W^{O},\\ {\mathrm{head}}_{i}=\mathrm{Attention}\left(QW_{i}^{Q},KW_{i}^{K},VW_{i}^{V}\right), \end{array}$$

where the learned linear projections are parameter matrices $W_{i}^{Q}\in R^{d\times d}$, $W_{i}^{K}\in R^{d\times d}$, and $W_{i}^{V}\in R^{d\times d}$, and the concatenation projection is parameter matrix $W^{O}\in R^{d\times d}$.

There is a challenge in directly implementing the above multihead attention mechanism into the graph pooling operation: while the multihead attention mechanism considers the internal connections between nodes, the linear projection of keys and values still inhibits the improvement in the attention. For this reason, inspired by DiffPool [21], we employed GNN models to generate keys and values in the multihead attention mechanism, such that it jointly considers the graph structure and the internal connections between nodes. Given the input node features $H^{k}\in R^{n^{k}\times d}$ and coarsened adjacency matrix $A^{k}\in R^{n^{k}\times n^{k}}$ at layer *k*, the inputs of keys and values in the multihead attention can be generated as follows:

(7) $$\begin{array}{c} \mathrm{Keys}={\mathrm{GNN}}_{k,\mathrm{key}}\left(H^{k},A^{k}\right),\\ \mathrm{Values}={\mathrm{GNN}}_{k,\mathrm{value}}\left(H^{k},A^{k}\right). \end{array}$$

Note that the GNN models for keys and values can be employed based on specific tasks, and GCN [5], GraphSAGE [4], and GAT [17] all can adapt to the model. In contrast to the two GNNs in DiffPool that directly and linearly generate node embeddings and clustering assignments, separately, the two GNNs called key and value were designed to consider graph structure information in the graph multiheaded attention mechanism, which then enable the attention mechanism to learn the optimal node clustering assignments.

Based on the above ingredients, graph multihead attention (GMA) can be formally expressed as follows:

(8) $$\begin{array}{c} \mathrm{GMA}\left(Q,H,A\right)=\mathrm{Concat}\left({\mathrm{head}}_{1},\ldots ,{\mathrm{head}}_{h}\right)W^{O},\\ {\mathrm{head}}_{i}=\mathrm{Attention}\left(QW_{i}^{Q},{\mathrm{GNN}}_{k,\mathrm{key}}\left(H^{k},A^{k}\right),{\mathrm{GNN}}_{k,\mathrm{value}}\left(H^{k},A^{k}\right)\right), \end{array}$$

where $Q\in R^{n^{k+1}\times d}$ is a parameterized seed matrix that clusters the $n^{k}$ nodes into $n^{k+1}$ clusters.

In addition to GMA, following the transformer model architecture, each of the proposed pooling layer contains a fully connected feed-forward network (FFN), which is separately and identically applied to each row, followed by layer normalization. Thus, the overall architecture of graph multihead attention pooling (GMAP) can be formally described as follows:

(9) $$\begin{array}{c} \mathrm{GMAP}\left(Z\right)=\mathrm{LayerNorm}\left(Z+\mathrm{FNN}\left(Z\right)\right),\\ where\;Z=\mathrm{LayerNorm}(Q+\mathrm{GMA}(Q,H,A)). \end{array}$$

Note that the output of GMAP is the embeddings of the clusters in the coarsened graph, which serve as the node embeddings of the next graph convolutional layer. After compressing the nodes of the original graph into clusters, which are nodes in the coarsened graph, we need to reduce the adjacency matrix of the original graph to another refined adjacency matrix of the coarsened graph.

### 3.4. Coarsened Graph Construction

While a coarsened graph can be constructed by compressed clusters, the connections between nodes in the coarsened graph still need to be generated by the node clustering assignment matrix. We first consider the formation for node clustering assignment matrix by the attention function as in Equation (5). In this attention function, Q, K, and V denote the queries of clusters, the keys of nodes, and the values of nodes, respectively. The attention function in Equation (5) can be decomposed as follows:

(10) $$\mathrm{Attention}\left(Q,\;K,\;V\right)=\mathrm{softmax}\left(\frac{QK^{⊤}}{\sqrt{d}}\right)\times V,$$

where the first term $\mathrm{softmax}\left(\frac{QK^{⊤}}{\sqrt{d}}\right)$ is the scaled dot product of cluster queries with all keys of nodes followed by a softmax function, namely the weights on the corresponding values of nodes. Specifically, the dot product $QK^{⊤}$ denotes the correlation between the clusters and nodes. The function softmax(·) normalizes the correlation to the correlation weights. Thus, the first term is essentially a generated soft cluster assignment matrix S:

(11) $$S=\mathrm{softmax}\left(\frac{QK^{⊤}}{\sqrt{d}}\right).$$

Note that compared with the cluster assignment directly generated by GNNs in DiffPool [21], our proposed model can be considered as explicitly learning the data-dependent $n_{c}$ cluster centroids by specific learnable queries Q, and then using the queries Q and keys K to generate the cluster assignment.

For each pair of nodes in the coarsened graph, the edges are constructed by considering the edges between the corresponding clusters in the original graph. Therefore, we can formally construct the adjacency matrix of the coarsened graph as follows:

(12) $$A^{k+1}={S^{k}}^{⊤}A^{k}S^{k}.$$

### 3.5. Self-Supervised Mutual Information Module

Our pooling method, GMAP, can achieve hierarchical representation of the original graph. To discriminate the cluster representations in the hierarchical graph, the self-supervised learning mechanism is utilized to maximize the mutual information between the cluster representation and the hierarchically global representation.

To generate the global representation of the hierarchical graph, we leveraged a readout function to summarize the node representation of the hierarchical coarsened graph into a fixed size graph level representation as follows:

(13) $$r^{k}=\mathrm{Readout}\left(H^{k}\right)=\sigma \left(\frac{1}{n^{k}}\sum_{p=1}^{n^{k}}H^{k}\left(p,:\right)\right),$$

where σ(·) is a sigmoid function, and $n^{k}$ denotes the number of nodes in the *k*th layer graph.

As a proxy for maximizing the mutual information between the cluster representations and hierarchically coarsened graph, a discriminator function is employed to measure the probability scores that represent the clusters contained within the hierarchical coarsened graph. We formally show the discriminator function as follows:

(14) $$D\left(H_{i,:}^{k},r^{k}\right)=\sigma \left({H_{i,:}^{k}}^{⊤}W^{D}r^{k}\right),$$

where σ(·) is a sigmoid function, $H_{i,:}^{k}$ denotes the embedding of node *i*, and $W^{D}\in R^{d\times d}$ is the linear parametric matrix.

For the self-supervised mutual information objective, we followed the intuitions in [28,43] and employed a noise-contrastive-type objective with a standard binary cross-entropy (BCE) loss between positive samples $H^{k}$ from the coarsened graph and the negative samples ${\tilde{H}}^{k}$ from another graph in the same batch. Therefore, the objective can be defined as follows:

(15) $$L_{MI}^{k}=\frac{1}{n_{pos}+n_{neg}}\left(\sum_{i=1}^{n_{pos}}E_{pos}\left[logD\left(H_{i,:}^{k},r^{k}\right)\right]+\;\sum_{j=1}^{n_{neg}}E_{neg}\left[logD\left({\tilde{H}}_{j,:}^{k},r^{k}\right)\right]\;\right),$$

where $n_{pos}$ and $n_{neg}$ denote the number of positive and negative examples, respectively. Note that, the mutual information objective of GMAPS is complementary to the graph classification objective, which enables the proposed pooling layer, GMAP, to focus on both hierarchical and global structural properties.

### 3.6. Computational Complexity

The space complexity of each graph pooling layer is $O(nn_{c})$, as it depends on the soft cluster assignment matrix $S\in R^{n\times n_{c}}$. The computational complexity of node clustering assignment is $O(n^{2}d+nd^{2})$, which is dominated by a multihead attention operation. The computational complexity of coarsened graph construction is $O(n^{2}n_{c}+nn_{c}^{2})$. Because the adjacency matrix is usually sparse, the computational complexity is reduced to $O(|E|n_{c}+nn_{c}^{2})$, where |E| is the number of nonzero edges in the adjacency matrix.

## 4. Experiments

To evaluate the performance of the proposed pooling model, GMAPS, GMAPS was compared with a collection of state-of-the-art GNN-based models on six benchmarks in terms of graph classification tasks. Furthermore, detailed ablation and parametric analyses were conducted to characterize the proposed pooling model.

### 4.1. Datasets

We adopted six public benchmark graph classification datasets among the TUDatasets [44]. The datasets are publicly available at https://chrsmrrs.github.io/datasets/docs/datasets/ (accessed on 22 June 2022). Statistics and properties are summarized in Table 1 with a detailed description as follows: D&D and PROTEINS are two protein graph datasets, MUTAG is a small molecule graph dataset, and the remaining ones are the social network datasets. Note that there are no node features in three social network datasets, and we encoded the node degrees into one-hot vectors as node features, which explicitly concerned the structural information.

### 4.2. Baselines

GMAPS was compared with state-of-the-art GNN-based models that could be categorized into two groups: global graph neural networks and graph pooling models.

Global graph neural networks include three representative models: GCN [5], GraphSAGE [4], and GAT [17], which learn node-level representations. Therefore, to achieve graph-level representations, we employed a readout function to summarize the learned node representations to a fixed-size graph representation.

For the graph pooling models, we deployed the same hierarchical GNN architecture, replacing only the pooling methods in it. To evaluate the performance of the proposed pooling model, gPool [22], ASAP [41], SAGPool [23], and DiffPool [21] were adopted as comparison methods. Specifically, gPool and SAGPool both feed the coarsened graph by a heuristic measure strategy to select a subset of nodes, the former by a trainable projection vector and the latter by attention between node features and graph topology. Furthermore, ASAP first considers the subgraphs within the fixed receptive field as clusters and then uses the self-attention among the local structure to measure the fitness scores for coarsened graphs. DiffPool learns a clustering assignment matrix with extra GNNs models to cluster nodes and to generate the adjacency matrix of the coarsened graph.

Finally, to further analyze the performance of the proposed pooling models, we introduced two variants: GMAP (Equation (9)) condenses the nodes to clusters with graph multihead attention mechanism, and GMAPS additionally employs self-supervised mutual information maximization to ensure the uniformity of the cluster representations in a hierarchical coarsened graph and the global representation of a coarsened graph.

### 4.3. Implementation Details

**Figure 2:**
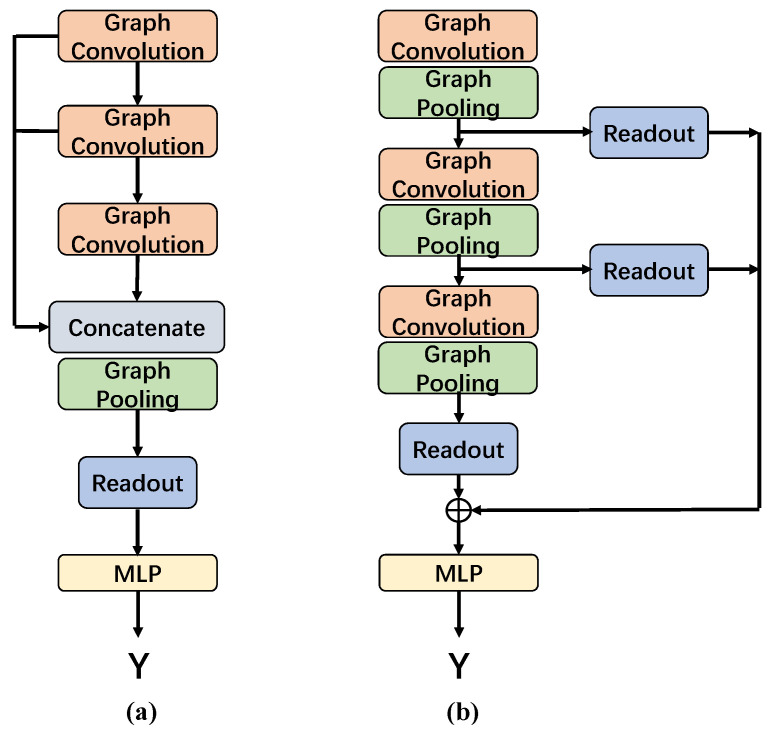
Illustration of architectures of (**a**) hierarchical GNN and (**b**) global GNN (GCN, GraphSAGE, and GAT).

Following many previous studies [21,45], for all baselines, 10-fold cross-validation was used to randomly split each dataset into training, validation, and test sets in a ratio of 80%/10%/10%. Then, we performed the randomly splitting process 10 times with 10 different seeds, and we report the average accuracy with standard deviation. For baseline methods, we implemented the source code released by PyTorch Geometric (PyG) [46], and the hyperparameters followed the default setting. To achieve a fair comparison, all baselines were implemented on top of the same GNN architecture. In our study, the hierarchical GNN architecture consisted of three convolutional layers, two pooling layers, a readout module, and an MLP module. Two pooling layers were interspersed between convolutional layers, separately, and the jumping knowledge strategy [47] was employed to concentrate the hierarchical representations generated by each convolutional layer. The MLP model consisted of three fully connected layers and a softmax classifier. For three global GNN models (GCN, GraphSAGE, and GAT), the GNN architecture consisted of three corresponding convolutional layers, followed by a global mean readout function. The architectures are illustrated in Figure 2.

For all datasets, the learning rate was set to $5\times 10^{-4}$, and weight decay was set to $1\times 10^{-4}$. Because D&D has a large number of average nodes, its hidden size and batch size were discreetly set to 32 and 10, respectively. For the other datasets, the hidden size was set to 128, and batch size ws set to 128. The pooling ratio was set to 25% for all baselines. For the MLP, the hidden size of the three fully connected layers were separately set to 128 and 56, the number of graph classification labels, and the dropout ratio was set to 50%. Furthermore, early stopping was implemented with a patience parameter of 50, where the training process stopped if the performance on the validation set did not improve beyond 50 epochs.

### 4.4. Graph Classification

The results of graph classification are reported in Table 2. As we can see, GMAPS consistently outperformed all baselines on all datasets, and GMAP outperformed other baseline methods or achieved comparable performance to the other baseline methods. For instance, GMAPS achieved 9.5% and 13.8% improvement on D&D and MUTAG datasets over GCN without hierarchical pooling, respectively. These results demonstrate that the proposed hierarchical pooling strategies are powerful and versatile.

For global GNN baseline methods without hierarchical pooling, such as GCN, GraphSAGE, and GAT, their overall performance was weaker than that of the hierarchical graph pooling approaches, especially on D&D and MUTAG datasets. For such bioinformatic networks, the structure of the subgraphs therein often represents different properties of the molecule and is crucial for learning the graph representations. Global GNN baseline methods only globally summarize the node representations and ignore the graph structural information. Thus, they struggle to achieve satisfactory performance.

Among the hierarchical graph pooling models, DiffPool achieved superior results in most cases, while the other baseline methods had their own performance on various datasets. We argue that the major reason is that their respective heuristic node selection strategies are only applicable to specific scenarios and are not versatile. Specifically, DiffPool utilizes an auxiliary link prediction objective during training to cluster the nearby nodes. In contrast, our proposed model considers both the graph structure and the internal connections between nodes; the satisfactory performance on different datasets further validates the generality of GMAPS. Note that the performance on D&D, PROTEINS, and MUTAG showed an impressive improvement over that of the global baseline method, while the performance on IMDB-B, IMDB-M, and COLLAB was similar to that of the baseline method. This may be due to the fact that the latter three social network datasets lack raw features and encode node degrees as features.

Finally, among our proposed models, GMAPS, which employs self-supervised mutual information maximization, consistently outperformed GMAP, which indicated the mutual information maximum module is facilitative for graph pooling in the graph classification task.

### 4.5. Graph Reconstruction

**Figure 3:**
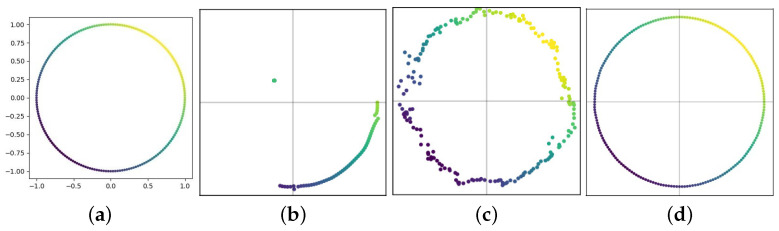
Reconstruction results of the ring graph: (**a**) original, (**b**) gPool (error = 1), (**c**) DiffPool (error = $10^{-2}$), and (**d**) GMAP (error = $10^{-6}$).

To quantity the amount of information about the graph retained by different pooling methods, we trained an autoencoder to reconstruct the input node features from the pooled representations. The learning objective to minimize the mean squared error between the original feature *X* and the reconstructed features $X^{rec}$ was defined as $||X-X^{rec}{\left|\right|}^{2}$. To upscale the coarsened graph back to its original graph, for node clustering methods (DiffPool and GMAP), we transposed the pooling operation as $X^{rec}=SX^{pool}$. For the top-K method (gPool), we used the unpooling operation proposed by [22]. We plot the visualization results in Figure 3 with auxiliary numerical measures (two-dimensional mean squared error). A good pooling method should recover the original graph as much as possible. It is obvious that gPool failed to recover the entire graph information because the top-K pooling operation dropped portions of the graph. The noisy result of DiffPool indicated a partial loss of information in the coarsened graph, while GMAP produced an almost perfect reconstruction result, which demonstrated its power in retaining meaningful information.

### 4.6. Ablation Studies and Visualization

As mentioned above, our proposed pooling models can be integrated into various GNN architectures. To explore their performance with different graph convolutional strategies, we implemented GraphSAGE and GAT, except for the default GCN convolutional layer. The experiments were conducted on the D&D, PROTEINS, and MUTAG datasets, and the results are presented in Table 3. As shown in Table 3, the graph classification performance depends not only on the pooling strategy but also on the choice of the graph convolutional strategy. In particular, GMAPS outperformed GMAP for any graph convolutional configuration on any dataset, which further confirmed the optimization of the self-supervised mutual information maximization module for the pooling layer in the graph classification task.

**Figure 4:**
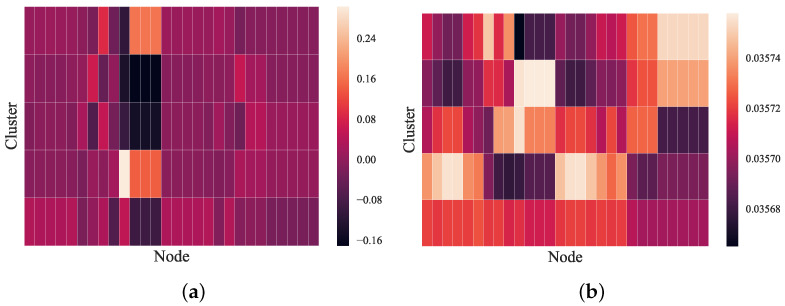
Visualization of the assignment matrices of DiffPool and GMAPS. Rows represent clusters in the coarsened graph, while columns are the nodes in the original graph: (**a**) DiffPool and (**b**) GMAPS.

To further explore the clustering assignment process of the pooling model, a visualization of the clustering assignment matrix *S* is presented in Figure 4. We randomly selected a graph in the MUTAG dataset to feed into the DiffPool and GMAPS models to obtain the assignment matrix in the first pooling layer. As shown in Figure 4a, DiffPool tended to generate a smooth coarsened graph through a dense clustering assignment matrix. In contrast, in Figure 4b, GMAPS has a relatively sparse cluster assignment matrix, which allows for distinct node features and graph structure in the coarsened graphs. The visualization once again demonstrated the satisfactory performance of GMAPS in capturing node features and graph topology.

### 4.7. Parameters Analysis

**Figure 5:**
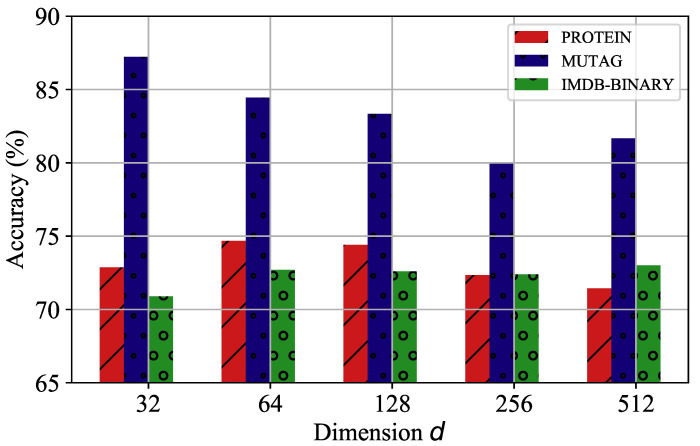
The parameter analysis of dimension *d*.

**Figure 6:**
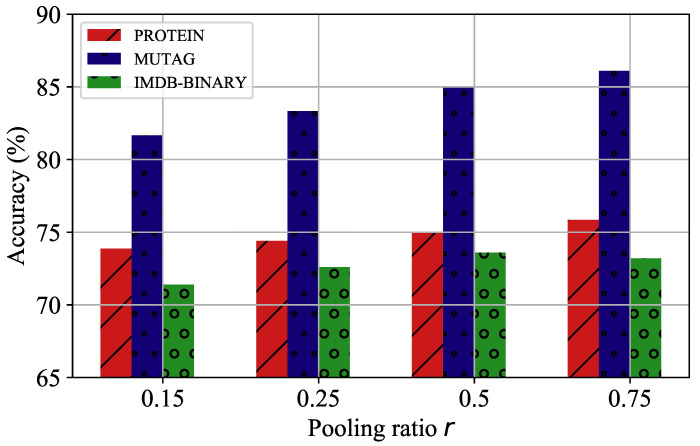
The parameter analysis of pooling ratio *r*.

We further investigated the effect of two hyperparameters (dimension *d* and pooling ratio *r*) on the pooling model, GMAPS, by considering the PROTEIN, MUTAG, and IMDB-BINARY datasets with various settings. Figure 5 shows that for the PROTEIN and IMDB-BINARY datasets, too large and too small dimensions diminished the accuracy, and 128 was the optimal choice. However, for the MUTAG dataset, due to its relatively simple graph number and structure, a lower dimension catered to its node representations, and the accuracy decreased instead as the dimension rose. The hyperparameter analysis of the pooling rate *r* is presented in Figure 6. It is shown that for the three experimental datasets, the accuracies improved with increasing pooling rate, which means that the pooling rate cannot be set too small; otherwise, most of the graph structure information will be lost, and, thus, the accuracy in the graph classification tasks will be hampered.

## 5. Conclusions

In this study, we found that existing graph pooling methods either cause the unnecessary loss of node features or do not adaptively learn an accurate graph representation in a specific downstream task. To address these limitations, we designed a novel graph pooling operation called GMAPS, which can compress nodes into a coarsened graph in a soft clustering manner. It also utilizes self-supervised mutual information maximization to ensure consistency between the cluster representations and the hierarchical coarsened graph representation. To validate the performance of the proposed pooling operation, we conducted experiments on six publicly available datasets and compared the results with those of seven state-of-the-art baseline methods. The results of graph classification and graph reconstruction tasks showed the excellent performance of the proposed model. Considering that the proposed pooling approach is generally applicable to various graph-learning tasks, which are growing more crucial, we are certain that it will have a significant practical impact. In the future, we will attempt to employ local subgraphs or anonymous random walks to capture the coarsened graph’s structure for more accurate pooling operations because improved local subgraph property extraction can improve the coarsened graph’s accuracy and interpretability. The key challenge of this effort is the capture and representation of task-related local subgraphs’ properties, which will be the focus of our future study. In addition, applying self-supervised learning without contrastive pairs to graph classification tasks is another aim for future research.

## Figures and Tables

**Table 1:** Statistics of the datasets.

Dataset	|G|	Avg.|V|	Avg.|E|	|Y|
D&D	1178	284.32	715.66	2
PROTEINS	1113	39.06	72.82	2
MUTAG	188	17.93	19.79	2
IMDB-Binary	1000	19.77	96.53	2
IMDB-Multi	1500	13.00	65.94	3
COLLAB	5000	74.49	2457.78	3

**Table 2:** Results of graph classification in terms of average accuracy ± standard deviation.

Method	D&D	PROTEINS	MUTAG	IMDB-B	IMDB-M	COLLAB
GCN-mean	71.96 ± 4.78	73.24 ± 3.62	70.55 ± 10.25	72.51 ± 3.91	51.13 ± 3.16	80.51 ± 1.39
SAGE-mean	72.13 ± 2.62	71.44 ± 3.88	68.88 ± 15.15	72.21 ± 2.63	49.81 ± 3.93	79.66 ± 1.49
GAT-mean	71.36 ± 4.31	72.61 ± 4.91	69.44 ± 14.95	72.59 ± 2.83	50.46 ± 4.31	79.36 ± 1.67
gPool	74.87 ± 3.82	72.52 ± 3.81	72.77 ± 9.76	71.91 ± 3.36	50.26 ± 3.33	79.11 ± 2.15
ASAP	72.82 ± 3.17	71.17 ± 4.81	80.55 ± 10.01	72.51 ± 4.29	50.21 ± 5.23	77.52 ± 2.38
SAGPool	70.59 ± 3.11	70.36 ± 3.81	78.33 ± 8.76	70.41 ± 5.93	51.26 ± 3.59	78.89 ± 1.63
DiffPool	77.35 ± 3.41	72.97 ± 5.76	77.77 ± 9.29	70.59 ± 4.65	50.66 ± 3.67	79.43 ± 1.42
GMAP	77.61 ± 3.41	74.23 ± 4.32	81.67 ± 11.12	72.09 ± 4.01	51.33 ± 4.67	80.72 ± 1.38
GMAPS	**78.81 ± 4.05**	**74.41 ± 3.58**	**83.33 ± 8.95**	**72.61 ± 4.38**	**51.67 ± 5.41**	**80.97 ± 1.41**

**Table 3:** Results of GMAP and GMAPS with various convolutional strategies.

Method	D&D	PROTEINS	MUTAG
GMAP-GCN	77.61 ± 3.41	74.23 ± 4.32	81.67 ± 11.12
GMAP-GraphSAGE	76.92 ± 3.48	72.07 ± 4.36	80.42 ± 10.58
GMAP-GAT	76.79 ± 4.15	72.97 ± 4.91	80.11 ± 11.62
GMAPS-GCN	78.81 ± 4.05	74.41 ± 3.58	83.33 ± 8.95
GMAPS-GraphSAGE	78.63 ± 3.11	72.97 ± 3.75	81.96 ± 9.72
GMAPS-GAT	78.37 ± 3.19	73.87 ± 4.88	81.25 ± 10.7

## Data Availability

The data presented in this study are openly available in an open access repository at [44].
